# A Window to the Brain—The Enduring Impact of Vision Research

**DOI:** 10.3390/brainsci15050453

**Published:** 2025-04-26

**Authors:** George Ayoub

**Affiliations:** Psychology Department, University of California, Santa Barbara, CA 93106, USA; ayoub@psych.ucsb.edu

**Keywords:** retina, vision, neurochemistry, neuroanatomy, neurophysiology, neurodevelopment, neurological health

## Abstract

The visual system has served as an expeditious entry point for discerning the mechanism of action of many brain systems, spearheading multiple fields of neuroscience in the process. It has additionally launched the careers of countless scientists, as we have crafted new means to understand neuronal structures and their functions, leading to advances in many areas of the sciences. Indeed, one can readily mark the onset of the scientific examination of the visual system with the 1851 invention of the ophthalmoscope by Hermann von Helmholtz, and the trichromatic theory of color vision in 1802. The Young–Helmholtz understanding the red–green–blue nature of color vision became the foundation to understanding sensory system function that visual artists and also contemporary flat panel displays rely on. It is fascinating to realize that the paintings of Georges Seurat and an iPhone display share a commonality of this application of the trichromatic theory. While it was not until 1956 that the existence of cells responsive to three different ranges of wavelengths was proven with the work of Gunnar Svaetichin, this proof in many ways marked the advancement of tools to visualize at a microscopic level, a full century after the Young–Helmholtz theory was developed. Just a decade later, in 1966, the person widely considered as the founder of modern neuroscience, Stephen Kuffler, founded the Harvard neurobiology department. It was from Kuffler’s work with his post-doctoral students that many new fields of study were created and from whom many of the neuroscience programs across the US were founded. In terms of the visual system, Kuffler and his team were key in detailing areas of retinal neuroanatomy, neurochemistry, neurophysiology, and developmental neurobiology. This paper traces areas in visual system research that provide our understanding of the disparate areas of brain sciences. As such, there are six categories that are evaluated, each of which spawned work in multiple areas that have become mainstays in neuroscience. These range from fields that were dominant a half century ago to ones that have their origins in this decade. The commonality is that all of these owe their origin to Helmholtz and Kuffler, polymaths of the nineteenth and twentieth centuries. We will examine the impact of vision research across the following fields of neuroscience: sensory system function, neuroanatomy, neurochemistry, neurophysiology, developmental neurobiology, and neurological health and disease.

## 1. Introduction

Vision research has long served as a crucial gateway for understanding the complex mechanisms of the brain. The eye, particularly the retina, acts as both a sensory organ and a model system to explore fundamental neural processes. Insights gained from studying the visual system have significantly advanced our knowledge of neuroanatomy, neurochemistry, sensory system functions, neurophysiology, developmental neurobiology, and neurological health and disease. This chapter elucidates how findings from vision research have contributed to our broader understanding of brain function.

### 1.1. A Window to the Brain

The eye is often referred to as a “window to the brain”, and this analogy is particularly pertinent in neuroscience. The retina contains specialized neurons that transduce light into electrical signals, making it an accessible site for studying neural processing. Techniques such as electrophysiology and imaging have allowed researchers to investigate the processing of visual information, revealing fundamental principles of neural coding and plasticity that are applicable to other sensory modalities and cognitive functions.

### 1.2. Retinal Structure and Function

The layered structure of the retina, comprising photoreceptors, bipolar cells, and ganglion cells, serves as a microcosm for understanding broader neural circuits. Research on retinal processing has illuminated how information is encoded and transmitted to the brain, highlighting the principles of parallel processing and spatial resolution that are fundamental to neural networks throughout the central nervous system.

### 1.3. Neuroanatomy

Vision research has significantly contributed to our understanding of neuroanatomical organization. Studies of the visual pathways, from the retina to the primary visual cortex (V1) and beyond, have provided insights into the organization and connectivity of neural circuits.

### 1.4. Visual Pathways

The retinofugal pathway, comprising the optic nerve, optic chiasm, and lateral geniculate nucleus (LGN), exemplifies how visual information is relayed and processed. The segregation of inputs from different types of photoreceptors and ganglion cells has informed our understanding of how the brain interprets color, motion, and depth.

### 1.5. Cortical Mapping

Research using techniques such as fMRI and electrophysiology has helped map the cortical areas involved in visual processing, revealing the hierarchical organization of visual information processing from V1 to higher-order visual areas. These findings underscore the importance of the visual system as a model for exploring functional specialization in the brain.

### 1.6. Neurochemistry

The study of the visual system has also provided insights into the neurochemical underpinnings of neural function. Key neurotransmitters, such as glutamate and GABA, play critical roles in retinal signaling and have been shown to influence neural plasticity and adaptation in the brain.

### 1.7. Neurotransmitter Systems

Research on neurotransmitter pathways in the retina has informed our understanding of similar systems in the brain. For instance, the role of dopamine in modulating retinal processing has parallels in the modulation of cortical activity, emphasizing the interconnectedness of neurochemical systems across different brain regions.

### 1.8. Sensory System Functions

The visual system’s complexity serves as a model for elucidating general principles of sensory processing. The study of visual perception has provided insights into how sensory systems integrate information from the environment, leading to coherent perceptual experiences.

### 1.9. Visual Perception and Processing

Investigations into phenomena such as visual illusions and perceptual constancy have revealed how the brain constructs a stable representation of the visual world. These principles extend to other sensory modalities, contributing to a comprehensive understanding of sensory integration and perception.

### 1.10. Neurophysiology

The neurophysiological aspects of vision research have been instrumental in elucidating the dynamics of neural activity. Techniques such as single-cell recording and calcium imaging have advanced our understanding of how neurons communicate and process information in real time.

### 1.11. Neural Encoding and Decoding

Research on the encoding of visual stimuli in the retina and visual cortex has led to the development of models that describe how sensory information is transformed into neural firing patterns. These models have implications for understanding neural coding across various sensory systems, enhancing our grasp of brain function.

### 1.12. Developmental Neurobiology

Vision research has provided valuable insights into developmental neurobiology, particularly regarding how sensory experiences shape neural development and plasticity.

### 1.13. Critical Periods

Studies have shown that the visual system undergoes critical periods of development, during which sensory input is essential for proper neural circuit formation. These findings have profound implications for understanding neurodevelopmental disorders and the timing of interventions.

### 1.14. Neurological Health and Disease

Understanding the visual system has significant implications for neurological health and disease. Vision research has informed the pathophysiology of various neurological conditions, including neurodegenerative diseases, sensory processing disorders, and traumatic brain injuries.

### 1.15. Disease Mechanisms

Investigations into retinal degeneration and other ocular diseases have provided insights into the mechanisms of neural cell death and regeneration. The retina serves as a model for studying neurodegenerative processes in the brain, allowing for the development of therapeutic strategies that may be applicable to other regions of the central nervous system.

### 1.16. Biomarkers and Interventions

The retina’s accessibility makes it an ideal site for identifying biomarkers for various neurological conditions. Advances in retinal imaging techniques have enabled the early detection of diseases such as Alzheimer’s and Parkinson’s, highlighting the potential for integrating vision research with broader neurological assessments.

The contributions of vision research to our understanding of brain mechanisms are profound and multifaceted. By serving as a model system, the visual system has provided critical insights into neuroanatomy, neurochemistry, sensory processing, and neurodevelopment, while also informing our understanding of neurological health and disease. As research continues to advance, the intersection of vision and brain science promises to reveal even deeper insights into the workings of the human brain.

## 2. Sensory System Transduction and Psychophysics

The study of sensory systems through the lens of the eye has provided profound insights into how we perceive the world around us. Pioneering work by researchers such as Ewald Hering, Hermann von Helmholtz, and David Hubel and Torsten Wiesel has shaped our understanding of visual processing, particularly in relation to retinal transduction mechanisms, color perception, and contrast sensitivity.

### 2.1. Retinal Transduction Mechanism

The retinal transduction mechanism is the process by which photoreceptors convert light into electrical signals, called receptor potentials, which directly influence the rate of neurotransmitter release. This process begins when photons are absorbed by opsin photopigments in the photoreceptors (rods and cones), leading to a biochemical cascade that ultimately results in hyperpolarization of the photoreceptor cell. This hyperpolarization decreases the release of the neurotransmitter glutamate from the synaptic terminals, the first step in signaling to bipolar and horizontal cells [1,2].

### 2.2. Phototransduction

Rods: Highly sensitive to light, rods are responsible for vision in low-light conditions. They contain the photopigment rhodopsin, which undergoes a conformational change upon photon absorption, initiating the phototransduction cascade.

Cones: Responsible for color vision and high acuity, cones contain three types of photopigments sensitive to different wavelengths corresponding to red, green, and blue light (RGB). This Young–Helmholtz trichromatic theory of color vision posits that all visible colors are represented by varying the proportions of these three primary colors.

### 2.3. Color Perception and Contrast

The understanding of color perception has evolved significantly through the work of Hering and Helmholtz. Hering proposed the opponent-process theory, which suggests that color perception is controlled by opposing pairs of colors: red–green, blue–yellow, and black–white. This theory complements Helmholtz’s trichromatic theory by explaining phenomena such as color afterimages and color blindness [3,4].

RGB Color Model: The RGB color model is foundational in understanding how colors are perceived. It is based on the additive mixing of red, green, and blue light, which corresponds to the three types of cones in the retina. By varying the intensity of these colors, a wide spectrum of colors can be produced, allowing for rich visual experiences.

Color Contrast: Color contrast is essential for visual perception, enabling the differentiation of objects in various lighting conditions. The retina’s CENTER/SURROUND organization enhances contrast by allowing ganglion cells to respond differently to light in their center versus their surrounding regions. This mechanism is crucial for edge detection and spatial resolution in visual processing.

### 2.4. Visual Cortex

The work of David Hubel and Torsten Wiesel further elucidated the neural processing of visual information in the retina and beyond. Their research demonstrated how visual stimuli are processed in the primary visual cortex, revealing the organization of receptive fields and the importance of orientation selectivity in visual perception. Their findings have been instrumental in understanding how the brain interprets complex visual scenes.

The study of the eye and its sensory systems has provided invaluable insights into the mechanisms of vision, including retinal transduction, color perception, and contrast sensitivity. The foundational theories which first began with Helmholtz are the basis for all RGB color displays today, and explain why printed colors are the opposing or contrasting colors (CMY), because it is the color reflected off the printed color that is perceived, rather than the transmitted color from an RGB screen [5].

## 3. Neuroanatomy

The retinal synaptic structure is a highly organized network that plays a crucial role in visual processing. This structure is composed of five types of neurons, including photoreceptors, bipolar cells, ganglion cells, horizontal cells, and amacrine cells, in a regular array.

### 3.1. Photoreceptors and Their Synapses

Rod and cone photoreceptors are the first cells in the visual pathway. They convert light into electrical signals through a process called phototransduction. Rods are highly sensitive to light and are responsible for vision in low-light conditions, while cones are less sensitive but enable color vision and high acuity in bright light. Photoreceptors release glutamate as a neurotransmitter, which is continuously released in darkness and suppressed by light. The light transducing outer segment of the photoreceptors are embedded in the retinal pigmented epithelium (RPE), a tissue that in humans is darkly pigmented to absorb any stray or reflected photos, thus optimizing visual acuity (in nocturnal animals, this epithelium, called a tapetum, is reflective, dimming acuity but enhancing night vision). The metabolically active photoreceptor pigments are continuously regenerated via resupply from the RPE.

### 3.2. Bipolar Cells

Bipolar cells serve as the second-order neurons in the retina, receiving input from photoreceptors and transmitting signals to ganglion cells. They are classified into two main types based on their response to light: ON-bipolar cells, which depolarize in response to light increases, and OFF-bipolar cells, which hyperpolarize. The synaptic connections between photoreceptors and bipolar cells occur in the outer plexiform layer (OPL) of the retina, where several types of synapses are formed, including invaginating ribbon synapses and flat junctions.

### 3.3. Inner Plexiform Layer, Ganglion Cells, and Amacrine Cells

The inner plexiform layer (IPL) is where bipolar cells synapse with ganglion cells and amacrine cells. This layer is organized into two distinct sublaminae: sublamina a, which contains synapses from OFF-bipolar cells; and sublamina b, which contains synapses from ON-bipolar cells. This organization allows for the segregation of ON and OFF pathways, enabling the retina to process visual information regarding increases and decreases in light intensity separately.

### 3.4. Horizontal Cells and Outer Plexiform Layer

Horizontal cells play a critical role in modulating the signals between photoreceptors and bipolar cells in the outer plexiform layer. They provide lateral inhibition, which enhances contrast, sharpening visual signals. This interaction is essential for the processing of visual information, particularly in the context of spatial resolution and contrast sensitivity.

### 3.5. Functional Implications

The retinal synaptic structure allows for the parallel processing of visual information. The distinct pathways for ON and OFF signals enable the retina to encode various aspects of the visual scene, such as brightness and contrast. This organization is crucial for higher visual processing and contributes to the overall functionality of the visual system.

Retinal synaptic structure, characterized by its layered organization and diverse cell types, is fundamental to visual processing. Retinal neuroanatomy research has significantly enhanced our understanding of neuronal connectivity, including broadening the understanding of the varieties of synaptic structures, lateral inhibitory feedback, use of reciprocal synaptic connections, and the repeating matrix pattern of synapses in topically stratified neural tissue.

## 4. Neurochemistry

Retinal synaptic chemistry is a rich interplay of neurotransmitters and receptors that facilitates the transmission of visual signals from photoreceptors to ganglion cells. This process is vital for converting light stimuli into the initial receptor potentials and then the action potentials that the brain can use for perception. Key neurotransmitters involved in this process include glutamate and gamma-aminobutyric acid (GABA), among others.

### 4.1. Photoreceptors and Glutamate Release

Rod and cone photoreceptors are the primary sensory cells in the retina. They respond to light by undergoing a molecular modification that leads to hyperpolarization and a decrease in the release of glutamate. In darkness, photoreceptors are depolarized and release glutamate. Glutamate is stored in synaptic vesicles and released through a calcium-dependent mechanism when photoreceptors are depolarized by light decrease. This dark release is essential for signaling to horizontal cells and bipolar cells.

### 4.2. Bipolar Cells and Their Responses

Bipolar cells are the second-order neurons that receive glutamatergic input from photoreceptors. They express different types of glutamate receptors, which are crucial for their function. ON-bipolar cells primarily express metabotropic glutamate receptor 6 (mGluR6), which, upon binding glutamate, activates a signaling cascade that leads to depolarization in response to light increases. In contrast, OFF-bipolar cells express ionotropic receptors such as AMPA and kainate receptors, which respond to glutamate, leading to hyperpolarization with light increases. Research has been instrumental in elucidating these mechanisms and the distinct roles of ON and OFF pathways in visual processing. These two categories of glutamate receptors permit the creation of both the ON and the OFF responses to light. They reveal a synaptic pathway to segregate identical information (glutamate release) into two distinct pathways. These two opposing responses to the same neurotransmitter permit optimized responsiveness by creating signals to both increases in light and decreases in it [6,7].

### 4.3. GABA and Inhibitory Signaling

GABA, the primary inhibitory neurotransmitter in the retina, is released by horizontal cells and some amacrine cells. Horizontal cells provide lateral inhibition, which enhances contrast and sharpens visual signals. They release GABA onto photoreceptors and bipolar cells, modulating activity to sharpen the image signals by enhancing signals at light/dark boundaries. Amacrine cells, which also release GABA, play a critical role in integrating signals from bipolar cells and modulating the output to ganglion cells. This inhibitory signaling is essential for temporal processing and motion detection in the visual system [8].

### 4.4. Calcium Dynamics and Synaptic Plasticity

Calcium ions (Ca^2+^) are pivotal in retinal synaptic transmission. The influx of Ca^2+^ through voltage-gated calcium channels at synaptic terminals triggers the release of neurotransmitters, including glutamate and GABA. The regulation of calcium dynamics is crucial for maintaining synaptic efficacy and plasticity, allowing the retina to adapt to varying light conditions and visual stimuli. Research has shown that disruptions in calcium signaling can lead to visual impairments, highlighting its importance in retinal health.

The retinal synaptic chemistry, characterized by the interplay of excitatory neurotransmitters like glutamate and inhibitory neurotransmitters like GABA, is fundamental to visual processing. This work has significantly advanced our understanding of these complex interactions, revealing how they contribute to the intricate processing of visual information in the retina. Tools developed to document the type of neurotransmitter used and the receptor types employed in the retina have been essential in similar identification throughout the nervous system, with one striking case in particular of providing an essential understanding of neurotransmission during LTP in the hippocampus [9,10,11,12].

## 5. Neurophysiology

Retinal physiology encompasses the mechanisms by which the retina processes visual information, involving a complex interplay of various cell types, synaptic interactions, and biochemical pathways. Retinal neurophysiology examines the mechanisms by which the retina processes visual information through its neural pathways and signaling. Research contributions have significantly advanced our understanding of the ON and OFF pathways, the CENTER/SURROUND systems, and the neural processing of signals [6,13].

### 5.1. Synaptic Interactions

The retina contains several types of neurons, including bipolar cells, horizontal cells, and ganglion cells, which interact through synaptic connections. Bipolar cells receive input from photoreceptors and transmit signals to ganglion cells. They can be classified into ON and OFF types based on their response to light. ON-bipolar cells depolarize in response to light increments due to the activation of metabotropic glutamate receptors (mGluR6), while OFF-bipolar cells depolarize in response to light decrements through ionotropic glutamate receptors.

### 5.2. Neural Processing of Signals

The retina processes visual signals through a series of interactions among various cell types, including photoreceptors, bipolar cells, amacrine cells, and ganglion cells.

Signal Integration: Bipolar cells integrate signals from multiple photoreceptors and horizontal cells, while amacrine cells modulate the output from bipolar cells before it reaches ganglion cells. This integration allows for the processing of temporal and spatial aspects of visual stimuli, such as motion detection and contrast enhancement.

Diversity of Ganglion Cells: The output from the retina is conveyed to the brain via ganglion cells, which can be classified into various types based on their response properties. This diversity allows the retina to encode different features of the visual scene, such as color, motion, and brightness.

### 5.3. Horizontal and Amacrine Cells

Horizontal cells provide lateral inhibition, enhancing contrast and spatial resolution in visual processing. They release GABA, which modulates the activity of photoreceptors and bipolar cells. Amacrine cells, which also release various neurotransmitters including GABA and glycine, play a crucial role in integrating signals from bipolar cells and modulating the output to ganglion cells. This integration is essential for processing temporal aspects of visual stimuli, such as motion detection.

### 5.4. Ganglion Cells and Signal Transmission

Ganglion cells are the final output neurons of the retina, transmitting visual information to the brain via their axons, which form the optic nerve. They receive input from bipolar and amacrine cells and can be classified into different types based on their receptive field properties and response characteristics. The organization of ganglion cells allows for the encoding of various aspects of the visual scene, including contrast, motion, and color.

### 5.5. ON and OFF Pathways

The retina encodes this visual information through two primary pathways: the ON and OFF pathways.

ON Pathway: This pathway is activated by increases in light intensity. ON bipolar cells, which express metabotropic glutamate receptor 6 (mGluR6), hyperpolarize in response to glutamate released by photoreceptors in darkness. When light is detected, photoreceptors hyperpolarize, leading to reduced glutamate release, which allows ON bipolar cells to depolarize and transmit signals to ON ganglion cells.

OFF Pathway: Conversely, the OFF pathway is activated by decreases in light intensity. OFF bipolar cells, which express ionotropic glutamate receptors (such as AMPA receptors), depolarize in response to the continuous release of glutamate in darkness. When light is present, the reduction in glutamate leads to the hyperpolarization of OFF bipolar cells, which then transmit signals to OFF ganglion cells.

This dual pathway system allows the retina to effectively encode both increases and decreases in light, contributing to contrast detection and overall visual perception.

### 5.6. CENTER/SURROUND Systems

The CENTER/SURROUND organization is a fundamental aspect of retinal processing that enhances spatial contrast and edge detection.

CENTER/SURROUND Organization: Retinal ganglion cells exhibit receptive fields that are organized into a central region (the “center”) and a surrounding region (the “surround”). The center typically responds to light in a specific manner (either ON or OFF), while the surrounding region responds in the opposite manner. For example, an ON-center ganglion cell will increase its firing rate when light is presented in the center of its receptive field and decrease its firing rate when light is presented in the surround.

Role of Horizontal Cells: Horizontal cells contribute to this organization by providing lateral inhibition. They release GABA, which inhibits the activity of neighboring photoreceptors and bipolar cells, thereby enhancing the contrast between the center and surround regions of the receptive field. This mechanism is crucial for detecting edges and improving visual acuity [8].

The physiology of the retina involves intricate mechanisms that enable the processing of visual information through ON and OFF pathways, CENTER/SURROUND systems, and intricate neural interactions. Retinal neurophysiologist provided critical insights into these processes, providing a framework for how sensory systems shape their signals to the brain cortexes, facilitating the cortical perceptive functions [14,15,16,17].

## 6. Developmental Neurobiology

The study of vision has significantly advanced our understanding of developmental neurobiology, particularly regarding the mechanisms of neural development and plasticity in the visual system. Pioneering work has given us the central parameters of developmental neurobiology, including the elucidation of the existence and role of critical periods in development, while other work has defined how the organization of visual fields in the lateral geniculate nucleus (LGN) and visual cortex are established from before birth.

### 6.1. Critical Periods in Development

Critical periods are defined as specific windows of time during which the nervous system exhibits heightened sensitivity to environmental stimuli, leading to significant changes in neural circuitry. These periods are essential for the proper development of neural systems, as first documented with vision [5].

Demonstration in Visual Cortex: Wiesel and Hubel’s groundbreaking studies in the 1960s demonstrated critical periods in the visual cortex of kittens. They found that monocular deprivation (closing one eye) during a specific developmental window resulted in permanent deficits in visual acuity and altered ocular dominance in the visual cortex. This work established that these critical periods occur, during which visual experience is necessary for the normal development of visual pathways [17].

### 6.2. The Development of Visual Fields in the LGN

The LGN serves as a relay station for visual information from the retina to the visual cortex. The development of visual fields in the LGN is guided by retinotopic projections, which are organized spatially according to the layout of the retina.

Retinotopic Projection: Retinal ganglion cells project to the LGN in a manner that preserves the spatial organization of the visual field. This retinotopic mapping ensures that adjacent areas of the retina correspond to adjacent areas in the LGN, facilitating accurate visual processing.

Role of Random Stimuli: Research by Shatz and coworkers has shown that spontaneous neural activity and random visual stimuli during early development play a critical role in shaping the retinotopic maps in the LGN. These random stimuli help refine synaptic connections and establish the precise topographic organization necessary for effective visual processing [18].

### 6.3. Implications for Neurodevelopment of Pathways

The findings regarding critical periods and retinotopic mapping have profound implications for understanding neurodevelopmental pathways. They suggest the following:

Experience-Dependent Plasticity: The brain’s ability to adapt and reorganize itself is particularly pronounced during critical periods. This plasticity is essential for the establishment of functional neural circuits that are responsive to sensory input.

Potential for Interventions: Understanding the timing and mechanisms of critical periods can inform therapeutic strategies for addressing visual impairments and other neurodevelopmental disorders. For instance, interventions during critical periods may enhance recovery from sensory deprivation or injury.

The study of vision has provided key insights into developmental neurobiology, particularly regarding the essential nature of critical periods, the organizational development of visual fields in the LGN, and the implications for neurodevelopmental pathways [19,20].

## 7. Neurological Health and Disease

### 7.1. Using Retinal Vasculature to Predict/Understand Cardiovascular Health

The use of the retinal vasculature as a predictive tool for cardiovascular health has been instrumental in the early diagnosis of cardiovascular disease (CVD). The retina, being a direct extension of the central nervous system, with a readily imaged network of blood capillaries, offers a unique window into the vascular health of an individual. The non-invasive nature of retinal imaging allows for the assessment of microvascular changes that correlate with systemic vascular conditions, including CVD, as first documented decades ago [21,22].

### 7.2. Oculomics and Cardiovascular Disease

Flammer’s research emphasizes the concept of systemic health being observable via retinal vasculature. This has been given the name “oculomics”, whereby the eye provides a window to the health status of multiple body systems. The retinal vascular network can reveal critical information about systemic vascular status, as abnormalities in retinal microvasculature have been linked to increased cardiovascular risk, mortality, and various risk factors such as hypertension and diabetes. Recent advancements in imaging technologies, such as optical coherence tomography angiography (OCT-A) and machine learning algorithms, have enhanced the ability to quantify retinal vascular parameters, making them valuable biomarkers for cardiovascular health [23].

The application of artificial intelligence (AI) systems in analyzing retinal images has shown promise in predicting cardiovascular events and stratifying risk more effectively than traditional methods. Studies have demonstrated that AI algorithms can identify features in retinal images that correlate with cardiovascular risk factors, thereby facilitating early intervention and management.

### 7.3. Expanding Applications: Glaucoma and Other Disorder Diagnoses

Building on the foundational work in cardiovascular risk assessment, researchers like Zee have begun to explore the potential of retinal imaging in diagnosing other conditions, such as glaucoma and autism. The integration of AI systems in this domain has opened new avenues for understanding and diagnosing these conditions through retinal vasculature analysis [24]. While Zee has identified retinal and neurodevelopmental disorders using AI, others are using AI of retinal images to identify Alzheimer’s and other dementias [25].

### 7.4. Glaucoma Diagnosis

Zee’s efforts focus on utilizing retinal images to identify biomarkers associated with glaucoma. The disease is characterized by changes in the optic nerve and retinal nerve fiber layer, which can be detected through advanced imaging techniques. AI algorithms are being developed to analyze these images for early signs of glaucoma, potentially leading to timely diagnosis and treatment. The ability to automate this process not only enhances diagnostic accuracy but also increases accessibility to screening in diverse populations [26].

### 7.5. Autism and Dementia Diagnoses

In a groundbreaking approach, researchers are investigating the use of retinal imaging to identify biomarkers for autism spectrum disorders (ASDs). The hypothesis is that certain microvascular changes in the retina may correlate with neurodevelopmental conditions. By employing AI to analyze retinal images, researchers aim to uncover patterns that could serve as diagnostic indicators for autism, thereby facilitating earlier and more accurate diagnoses. This innovative application of oculomics could revolutionize the way we approach the diagnosis of complex neurodevelopmental disorders [24].

The integration of retinal vasculature analysis into cardiovascular risk assessment and the exploration of its applications in diagnosing glaucoma and autism represent a significant advancement in medical imaging and AI. The work of Flammer and Zee highlights the potential of oculomics to transform our understanding of systemic health and disease. As research continues to evolve, the promise of retinal imaging as a diagnostic tool across various medical fields becomes increasingly evident [26,27,28,29].

Similarly, early diagnosis of dementia (including Alzheimer’s) has now been reported using recorded retinal health measures over time and follow-up to learn which subjects later developed dementia. The findings show that retinal health indicators in midlife (revealing diminished vascular health) are predictors of later dementia.^26^ This could allow underlying conditions for future neurodegenerative disorders to be identified and addressed well in advance of disorder symptoms becoming evident.

### 7.6. Cellular Remodeling

Cellular remodeling in the context of vision refers to the structural and functional changes that occur in retinal cells in response to various stimuli or injuries. This remodeling can be both adaptive and maladaptive, influencing the progression of neurological diseases.

Neuroplasticity: The retina exhibits a remarkable capacity for neuroplasticity, which is crucial for recovery from injury and adaptation to changes in visual input. Studies have shown that retinal ganglion cells can undergo remodeling in response to changes in their environment, which can be influenced by factors such as neurotrophic factors and inflammatory responses [30,31].

Pathological Remodeling: In conditions such as glaucoma, cellular remodeling can lead to the degeneration of retinal ganglion cells, contributing to vision loss. Understanding the mechanisms underlying these changes is essential for developing targeted therapies to protect and restore retinal function.

### 7.7. Neuroprotection

Neuroprotection in the visual system involves strategies aimed at preserving neuronal function and preventing cell death in response to injury or disease.

Protective Agents: Research has identified various neuroprotective agents that can mitigate damage to retinal cells. For instance, neurotrophic factors such as brain-derived neurotrophic factor (BDNF) have been shown to support the survival of retinal ganglion cells and promote their regeneration after injury.

Role of Vascular Health: Vascular health is closely linked to neuroprotection in the retina. Flammer’s work emphasizes the importance of ocular blood flow and its regulation in maintaining retinal health. Impaired blood flow can lead to ischemic conditions that exacerbate neuronal damage and contribute to diseases such as diabetic retinopathy and age-related macular degeneration [32].

### 7.8. Vascular Health

The health of the vascular system is critical for maintaining the integrity of the retina and overall neurological function.

Ocular Blood Flow: Flammer’s research highlights the significance of ocular blood flow in preventing retinal diseases. Adequate blood supply is essential for delivering nutrients and oxygen to retinal cells, and disruptions in this flow can lead to cellular stress and degeneration [33,34].

Systemic Implications: The vascular health of the eye can serve as an indicator of systemic health. Conditions such as hypertension and diabetes can manifest in the retina, providing insights into the overall vascular health of an individual. Monitoring retinal vascular changes can thus be a valuable tool in assessing neurological health.

The study of vision has illuminated critical aspects of neurological health and disease, particularly through the lenses of cellular remodeling, neuroprotection, and vascular health. This work has advanced our understanding of these processes, paving the way for potential therapeutic interventions aimed at preserving and restoring vision in the context of neurological disorders [33,34].

## 8. Conclusions

The eye has played a key role in the exploration of nervous system function. Indeed, work in the 19th century that determined the trichromatic basis of color vision later informed work on color management in the 20th and 21st centuries. The explanation of how sensory transduction works in photoreceptors has led to understanding transduction in other sensory systems. The early efforts to identify the neuroanatomy and neurochemistry of synaptic communication demonstrated the complexity and grand variety of synapses and circuitry. The detailed examination of neural processing in the retina has led to the understanding of neural networks. Learning how the visual system develops has spurred entirely new mechanisms to consider in the neurodevelopment of all brain systems. And it is the lessons learned from the retina in health and disease that has provided some of the greatest advances in health care, from the recognition that retinal vasculature is a window to cardiovasculature, to nutritional aspects for retinal health which inform us about such aspects throughout the brain, to the retinal systems involved in neuroprotection which are being found reproduced in other aspects of brain function. This long history of the most accessible part of the brain has been the gift that much of neuroscience has been built upon.

## Abbreviations

The following abbreviations are used in this manuscript:

LGN	Lateral Geniculate Nucleus
GABA	Gamma Amino Butyric Acid
AMPA	α-Amino-3-hydroxy-5-methyl-4-isoxazolepropionic acid
OCT-A	Optical Coherence Tomography Angiography
